# RAGE at Tumor Microenvironment. Looking at Tumor-associated Macrophages

**DOI:** 10.3779/j.issn.1009-3419.2015.12.02

**Published:** 2015-12-20

**Authors:** Fernando DELGADO-LÓPEZ, Armando ROJAS

**Affiliations:** Biomedical Research Labs., Medicine Faculty, Catholic University of Maule, 3605 San Miguel Ave., Talca, Chile

A compelling body of evidence has demonstrated that activation of the receptor for advanced glycation end-products (RAGE) is responsible for triggering an inflammatory response and being associated with many clinical entities, including diabetes, neurodegerative diseases, cardiovascular diseases and cancer^[1-4]^.

RAGE is expressed in many tumor cell types where its activation is strongly associated with tumor growth, cell migration and invasion, angiogenesis and resistance to apoptosis. Far beyond its role on some tumor cell activities, RAGE is also expressed in many tumors infiltrating cells and thus contributing to the inflammation-related tumorigenesis^[5, 6]^. Of note, tumor microenvironment represents a particular compartment where most cells not only express RAGE, but also produce many RAGE ligands.

One of these infiltrating tumor cells are macrophages. This particular and heterogeneous population of innate myeloid cells, may undergo a polarized activation process once they infiltrated into tumor stroma and thus rendering two distinct polarization states; the "classically activated" type 1 macrophages (M1) and the "alternative activated" type 2 macrophages (M2)^[7, 8]^. The M1 phenotype, can be induced by bacterial products and interferon-γ (IFNγ) and exerts a cytotoxic effect on cancer cells, while the M2 phenotype can be induced by IL-4/IL-13 and promotes tumor cell growth and vascularisation. Interestingly, tumor-associated macrophages (TAMs) constitute the predominant component of leukocytic infiltrate in many solid tumors. TAMs have the potential to contribute to the earliest stages of neoplasia, smoldering inflammation at tumor microenvironment (M1 phenotype) and then, as tumor growth up, they are dynamically converted towards a M2 phenotype and exert reduced cytotoxic activities, and promote tumor growth, angiogenesis and immune-suppression. We recently demonstrated that the alarmin HMGB1, which are abundantly expressed at tumor microenvironment, increased the protumoral activities of M2 macrophages by a RAGE-dependent mechanism, thus favoring invasion of tumor cells, the formation of new blood vessel network and the methaloproteinase-9 production^[9]^. All these activities were abrogated by RAGE-targeting knockdown.

At first glance, these results seem to be paradoxical, considering that first, RAGE activation is associated with an inflammatory and cytotoxic profile and secondly, M2 macrophages display a well-known reduced cytotoxic activity. However, RAGE downstream signaling in M2 macrophages has been drifted away from its classical pro-inflammatory cascade, just rendering a deactivated NFκB pathway.

Although among the different strategies proposed lately to fight cancer, re-education of tumour-associated macrophages from M2 to M1 phenotype seems to be very attractive and potentially a novel approach to cancer intervention from the theoretical point of view. However, there is an urgent need of a more in-deep understanding of cell signaling changes produced by the polarization process in TAMs.

## Conflict of Interest Statement

The authors declare that the research was conducted in the absence of any commercial or financial relationships that could be considered as a potential conflict of interest.
